# Comparative life‐history responses of lacewings to changes in temperature

**DOI:** 10.1002/ece3.70000

**Published:** 2024-07-18

**Authors:** Hanna Serediuk, John Jackson, Sanne Maria Evers, Maria Paniw

**Affiliations:** ^1^ Department of Conservation Biology and Global Change Estación Biológica de Doñana (EBD‐CSIC) Seville Spain; ^2^ State Museum of Natural History NASU Lviv Ukraine

**Keywords:** climate change, demography, life cycle, life history, Neuroptera

## Abstract

Insects play a crucial role in all ecosystems, and are increasingly exposed to higher in temperature extremes under climate change, which can have substantial effects on their abundances. However, the effects of temperature on changes in abundances or population fitness are filtered through differential responses of life‐history components, such as survival, reproduction, and development, to their environment. Such differential responses, or trade‐offs, have been widely studied in birds and mammals, but comparative studies on insects are largely lacking, limiting our understanding of key mechanisms that may buffer or exacerbate climate‐change effects across insect species. Here, we performed a systematic literature review of the ecological studies of lacewings (Neuroptera), predatory insects that play a crucial role in ecosystem pest regulation, to investigate the impact of temperature on life cycle dynamics across species. We found quantitative information, linking stage‐specific survival, development, and reproduction to temperature variation, for 62 species from 39 locations. We then performed a metanalysis calculating sensitives to temperature across life‐history processes for all publications. We found that developmental times consistently decreased with temperature for all species. Survival and reproduction however showed a weaker response to temperature, and temperature sensitivities varied substantially among species. After controlling for the effect of temperature on life‐history processes, the latter covaried consistently across two main axes of variation related to instar and pupae development, suggesting the presence of life‐history trade‐offs. Our work provides new information that can help generalize life‐history responses of insects to temperature, which can then expand comparative demographic and climate‐change research. We also discuss important remaining knowledge gaps, such as a better assessment of adult survival and diapause.

## Graphical abstract

This works compares life cycle responses of 62 lacewing species to temperature variation. It provides new information that can help generalize life‐history responses of insects to temperature, which can then expand comparative demographic and climate‐change research.

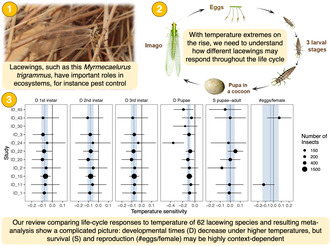

## 1 INTRODUCTION

Insects represent the most diverse group of animals, with over a million described species, accounting for more than half of all known living organisms (Chapman, 2006), and potentially making up over 90% of the animal species on Earth (Erwin, 1982; Wilson et al., 1999). They inhabit a wide range of terrestrial biomes and play a crucial role in all ecosystems, performing various functions such as soil turnover and aeration, dung burial, pest control, pollination, and wildlife nutrition. Changes in abundances and extinctions of insects have received widespread attention (Cardoso & Leather, 2019; Gossner et al., 2023; Lampert et al., 2023). Among the numerous factors contributing to declines in insect abundance, for example, land‐use changes (Newbold et al., 2020; Sala et al., 2000; Sánchez‐Bayo & Wyckhuys, 2019; Seibold et al., 2019; Settele et al., 2005), pesticide use (Gebert et al., 2022), or invasive species (Bates et al., 2020; Seebens et al., 2017; Wagner & Van Driesche, 2009), increases in temperature extremes under climate change play a particularly important role globally (Jackson, Johnson, et al., 2022; Jackson, Le Coeur, & Jones, 2022; Sánchez‐Bayo & Wyckhuys, 2019). This is because insects are ectotherms, and thus their physiology and, in turn, life‐history processes including growth, development, and reproduction are directly influenced by the temperature of their surrounding environment (Kobori & Hanboosong, 2017). The effects of temperatures on life‐history processes can be complex, and assessing the thermal biology across the entire life cycle of populations has been shown to be crucial to understand abundance changes (Radchuk et al., 2013). However, such assessments currently represent a large gap in the insect literature (Bowler & Terblanche, 2008; Carter & Sheldon, 2020; Kingsolver & Buckley, 2020).

Variation in temperature can have both sublethal and lethal effects on the physiological processes of insects (Ashbrook et al., 2024; Bowler & Terblanche, 2008; Feder et al., 1997; Morey et al., 2018). However, how these effects translate to changes in abundances and population fitness is often the result of differential impacts of temperature on different components of individual and population fitness, such as development, pupal mass, survival, and the accumulation of energy reserves. For instance, caterpillars of the European grapevine month (*Lobesia botrana*) have been shown to respond positively to higher temperatures in the context of climate change, showing faster development, increased survival rates, and improved evasion of natural enemies through “flee” behavior (Iltis et al., 2019). However, physiological trade‐offs in investment in different life‐history processes mean that faster development comes at a cost of a depletion of lipid reserves (−26%) and a reduced activity of enzymes that regulate immune responses (−34%) (Iltis et al., 2019). This can ultimately have adverse effects on survival, as fat stores are essential for enduring extended periods of food scarcity (Sinclair & Marshall, 2018). It may also carry over into the adult stage, having a detrimental impact on the dispersal and reproductive capabilities of adults (Vande Velde & Van Dyck, 2013).

The above example shows that understanding the effects of temperatures throughout the entire life cycle, and not just for particular life cycle stages or overall abundances, is critical to assess population‐fitness consequences of temperature variation (Carter & Sheldon, 2020). Most populations in natural communities, in ectotherms and endotherms alike, are structured by genetic and phenotypic traits, and ages or ontogenetic stages. In such structured populations, differential responses to the environment across life cycle stages, in part due to intrinsic physiological trade‐offs, may buffer populations from environmental change (Iltis et al., 2019; Radchuk et al., 2013; Villellas et al., 2015) or may even help populations adapt to climate change (Kearney et al., 2009). At the same time, persistence may be particularly threatened when abiotic factors such as temperature simultaneously reduce the survival and reproduction of several life cycle stages (Nakamura et al., 2016; Winkler et al., 2021).

While research on the effects of temperature on life‐history processes of insects is not new, a comprehensive comparison of how temperatures simultaneously affect development, survival, and reproduction across the life cycle in different species is thus far lacking (Ma et al., 2021). Our main aim is therefore to bridge this substantial knowledge gap, focusing on Neuroptera, commonly known as lacewings. Neuroptera stand out among other taxa due to relatively more available life‐history data, in particular in not peer‐reviewed (gray) literature, but remain underrepresented in comparative studies (Eickermann et al., 2023). In addition, Neuroptera play a crucial role as effective predators of various insect pests, such as mealybugs, mites, or aphids (Koutsoula et al., 2023; Nair et al., 2020; Yayla et al., 2020) and are an indicator of environmental health and ecosystem quality (Stelzl & Devetak, 1999; Verma et al., 2023). Here, we first perform a review of peer‐reviewed and gray literature to generate a database with responses to temperature and other key environmental drivers across the life cycle for Neuroptera. Secondly, we use the information generated in the review to perform a meta‐analysis and comparative life‐history analysis on the drivers of the covariation in life‐history responses to temperature across species.

## 2 MATERIALS AND METHODS

### 2.1 Neuroptera life cycle and thermal biology

Neuroptera is an insect order with complete metamorphosis. The typical developmental cycle for Neuroptera, with only a few exceptions, encompasses stages of egg, three larval stages (with some exceptions in Ithonidae), a pre‐pupa stage, pupa, and finally, the adult imago. The embryonic development in Neuroptera does not significantly differ from other taxa, with varying durations among species. Weight of egg decreases during embryogenesis. Hatching commences with embryonic molting and is a precarious period where the newly born larva is highly vulnerable. The short period as adult imago signifies the second crucial stage in Neuroptera development. During this time, the insect moves slowly and remains highly vulnerable. Within a few days, under favorable conditions, the adult reaches sexual maturity, mating takes place, and the female initiates egg laying.

During the egg, larvae, pupae, or adult stage of the life cycle, depending on the species, individuals can enter diapause, a period of suspended development (Canard, 2005; Canard & Volkovich, 2001). Diapause allows individuals to survive periods of unfavorable environmental conditions and to synchronize development and reproduction with favorable seasons. This condition is usually triggered by environmental cues such as changes in day length (photoperiod), temperature, and food availability. The life cycle stage in which diapause occurs and thermoregulation of how it is triggered vary relatively little within species, but can show marked differences among species (Nechols et al., 1987; Tauber et al., 1986). While the photoperiod‐induced diapause and lower temperature thresholds that trigger diapause have been extensively studied in Neuroptera, the effects of high temperatures on diapause remain unknown for most species. However, there is some evidence that high temperatures prolong diapause and increase mortality during diapause, as observed in *Micromus angulatus* (Potjomkina, 1987), *M. tasmaniae* (Syrett & Penman, 1981), and other species (Canard & Principi, 1984; Canard & Volkovich, 2001).

In addition to diapause, temperature affects egg development, larval growth rate, and pupation timing (Aghdam & Nemati, 2020; Pappas et al., 2013), but comparative studies on temperature responses across life cycle stages have not been performed. This is despite the fact that Neuroptera exhibit a wide range of temperature tolerance, allowing them to inhabit various environments—from temperate regions to tropical forests and even deserts (Canard, 2005). As poikilothermic organisms, they employ various behavioral adaptations to regulate body temperature and often select microenvironments that optimize their thermal conditions, such as areas with suitable temperature gradients or thermal buffers (Canard & Volkovich, 2001).

### 2.2 Literature review

We searched Web of Science and Scopus for literature for studies (published before March 31, 2023) that quantified the effects of temperature on different life‐history processes in Neuroptera as described in the previous section. Additionally, we used Google Scholar to search for articles, including gray literature, that may not have been indexed in the aforementioned databases. The latter included book chapters, monographs, or regional journals. We used the following search terms to find potentially suitable studies:

“(Neuroptera AND life cycle)” OR “(Chrysopidae AND life cycle)” OR “(Hemerobiidae AND life cycle)” OR “(Myrmeleontidae AND life cycle)” OR “(Coniopterigydae AND life cycle)” OR “(Sisyridae AND life cycle)” OR “(Ascaphidae AND life cycle)” “(Dilaridae AND life cycle)” OR “(Polystoechotidae AND life cycle)” OR “(Psychopsidae AND life cycle)” OR “(Coniopterygidae AND life cycle)” OR “(Ithonioidea AND life cycle)” OR “(Berothidae AND life cycle)” OR “(Mantispidae AND life cycle)” OR “(Nemopteridae AND life cycle)” OR “(Ascalaphidae AND life cycle)” OR “(Nemopteridae AND life cycle)” OR “(Nymphidae AND life cycle)” OR “(Osmylidae AND life cycle)” OR “(Nevrorthidae AND life cycle)” OR “(Sisyridae AND life cycle)” OR “(Neuroptera life cycle AND temperature)” OR “(Temperature AND Neuroptera development)” OR “(Temperature AND Chrysopidae development)” OR “(Temperature AND Hemerobiidae development)” OR “(Temperature AND Neuroptera life history)” OR “(Neuroptera life history)” OR “(Neuroptera temperature adaptation)” OR “(Climate change AND Neuroptera)” OR “(Climate change impact AND insects).”.

To be included in our final database, any study that matched our search terms had to quantitatively link temperature variation to life‐history processes, that is, life‐history processes were quantified under different temperatures. We discarded descriptive studies. We included studies performed in the field (in situ) as well as laboratory (in vivo) experiments. From the studies that matched our criteria to be included in the database, we recorded the species name, the location of their collection in as much detail as possible (continent, specific location, and coordinates if available). In cases where precise coordinates were absent but a specific location was indicated, we retrieved coordinates for the center of that location. We recorded the constant temperature used during the studies in degrees Celsius (°C) and, when available, the thermal constant “K,” which represents the thermal requirement for development and is measured in degree‐days (calculated by the equation K = (1/y) (x — t), where y = mean developmental rate, and x = temperature (°C)). If recorded in a study, we also noted how other abiotic and biotic factors affected life‐history processes under different temperatures, for example, food intake. Supplementary Material S1 provides a description and access to all articles/reports we extracted information from as well as details on the information collected.

For key life cycle stages (as detailed in *Neuroptera life cycle and thermal biology*), we recorded developmental times, survival rates, and reproductive rates in response to temperature. Developmental times included the duration (in days) of egg development; the three instar, the pupae, and the adult stage after emerging from the pupa and before egg‐laying; and of the reproductive period of females. We also recorded the average expected lifespan and generation time of adults in days. Reproduction included the percentage of females capable of reproducing and the total number of eggs laid by a single female. For survival, we recorded the percentage of successful larval emergence from eggs and the percentage of successful survival during the period from larva to adult. If articles provided minimum, maximum, and mean values, we also recorded these data as separate categories in our database. We also recorded the total number of individual insects used in an experimental treatment.

### 2.3 Meta‐analysis of temperature sensitivities across the life cycle

To compare the variation in life‐history processes (i.e., development times, survival, and reproduction) under different environmental regimes among species, we performed a meta‐analysis (Gurevitch et al., 2001). We first used the estimates of life‐history responses to different temperatures provided in the studies to fit effect sizes of temperature sensitives. For meta‐analysis, we restricted the raw dataset to include only studies with at least three observations for each life‐history process, and at least two different constant temperatures (typically in vivo or in situ treatments). We fit effect sizes of temperature sensitivities using univariate linear regression, where development times and reproduction (#eggs/female) were analyzed using log‐normal regression, and larval survival was analyzed using a logistic regression on survival percentage. The response variable in each regression was temperature in degrees Celsius. Then, for each regression, we extracted the coefficient for the temperature on the linear predictor scale (Z scores), which gave temperature sensitivity for each life‐history trait. We fit meta‐analysis models for the temperature sensitivities of each life‐history trait using the *meta* package, using meta‐analysis for correlation with direct use of our Z scores from linear regressions, and the sample size extracted from each study (total number of insects in calculations) (Balduzzi et al., 2019). Meta‐analysis models were fit using the inverse variance method, and we tested for heterogeneity using Cochran's Q analysis (Balduzzi et al., 2019).

### 2.4 Comparative analysis of lacewing life‐history strategies

To explore general patterns of among‐species differences in life‐history strategies independent of species temperature sensitivities, we analyzed the covariation in life‐history processes (i.e., development times, survival, and reproduction), after accounting for the effect of temperature and other confounding factors on this covariation (following Healy et al., 2019; Ozgul et al., 2023). To first account for the effects of covariates, we used multivariate generalized linear mixed effect models (Brommer et al., 2019), using the MCMCglmm package in R (version 4.1.2) (Hadfield, 2010). Multivariate models have been suggested as appropriate tools to make inferences based on disaggregated data in comparative studies (Clark et al., 2011). By using a hierarchical random‐effects model structure, the models also allowed us to include studies where it was not possible to calculate study‐specific variation for some life‐history processes in the meta‐analysis due to small sample sizes.

Our multivariate GLMMs modeled the covariation in life‐history processes as a function of temperature individuals were exposed to per treatment combination (in vivo or in situ). We also included a squared temperature term to allow for curvilinear responses. To improve convergence of the models, we standardized temperature values (mean = 0, SD = 1). We accounted for potential effects of regional bioclimatic factors affecting species by including *latitude* as a covariate in models (which is a proxy for bioclimatic conditions). None of the reviewed studies quantified temperature responses across the entire life cycle of Neuroptera. We therefore focused on response variables that were most consistently measured across studies (51 out of 274 records): developmental times of the three instar‐stages and pupae, survival of pupae to adult, and number of eggs per female. We performed an arcsine transformation on survival rates and log‐transformed the remaining response variables to ensure a Gaussian distribution of the model residuals (which improved model converge under the limited data). We also considered additional models with fewer covarying life‐history processes but a higher sample size to see whether our interpretation of life‐history strategies would change as we increased the number of species in the analyses. To do so, we fitted additional models in which we separately modeled developmental times (120 out of 291 records) and survival/reproduction (56 out of 291 records) as joint multivariate responses. All records that we used to fit covariation in six (developmental times, survival, and reproduction) and four (developmental times) life‐history processes were from peer‐reviewed studies. When modeling covariation in survival/reproduction only, records included gray literature, and we included *literature type* as a covariate (categorical, *yes* or *no* peer‐reviewed) in the MCMCglmm. The developmental times were mostly recorded under natural conditions (in situ; 114 out of the 120 records). We repeated the analyses removing the eight in vivo records, but the results did not change (Supporting Material S3).

In all models, the multivariate response was accounted for using a covariance matrix in which life‐history processes covaried within species. To incorporate the inherent nonindependent relationships among species as well as differences in temperature sensitivity, we used a random population (species × study combination) effect as variance term on the mean of life‐history responses and the slope of the temperature effect. We did not use the animal term in the MCMCglmm package to correct for phylogeny because the full analysis (51 records) consisted of nine species, including four species for which the phylogeny is not well resolved, while the analysis using developmental times (120 records) used 21 species but included 10 species for which phylogeny is not fully resolved (see Supporting Material S3 for all *R* scripts that include the modeling workflows).

The MCMCglmm package uses a Bayesian framework to fit models. We thus ran three independent chains using 50,000 iterations after a burn‐in of 10,000 iterations. We used a thinning interval of 25 steps, resulting in 2000 posterior parameter samples per chain. We determined model convergence using standard Bayesian checks (i.e., traceplots and Gelman–Rubin diagnostic; Brooks & Gelman, 1998). We considered differences in stage‐specific developmental times between temperature regimes to be significant when the 95% credible interval (C.I.) of the respective parameters did not overlap 0.

Lastly, we used the residuals of the multivariate model (observed – predicted mean value for each effect size) to investigate covariation among life‐history processes not accounted for by temperature or latitude effects. For this, we performed a varimax rotated principal component analysis (PCA) on the residuals, following Healy et al. (2019) and Ozgul et al. (2023). The residual variation in life‐history processes was scaled to l = 0 and SD = 1 to agree with PCA assumptions. After implementing the PCA, we used the Kaiser criterion (Legendre & Legendre, 2012) to explore how many axes sufficiently explain the variation observed in the data. This criterion is based on keeping only principal component axes with associated eigenvalues >1. We ran all analyses in R version 4.1.2 (R Core Team, 2021).

## 3 RESULTS

### 3.1 Literature review

**FIGURE 1:**
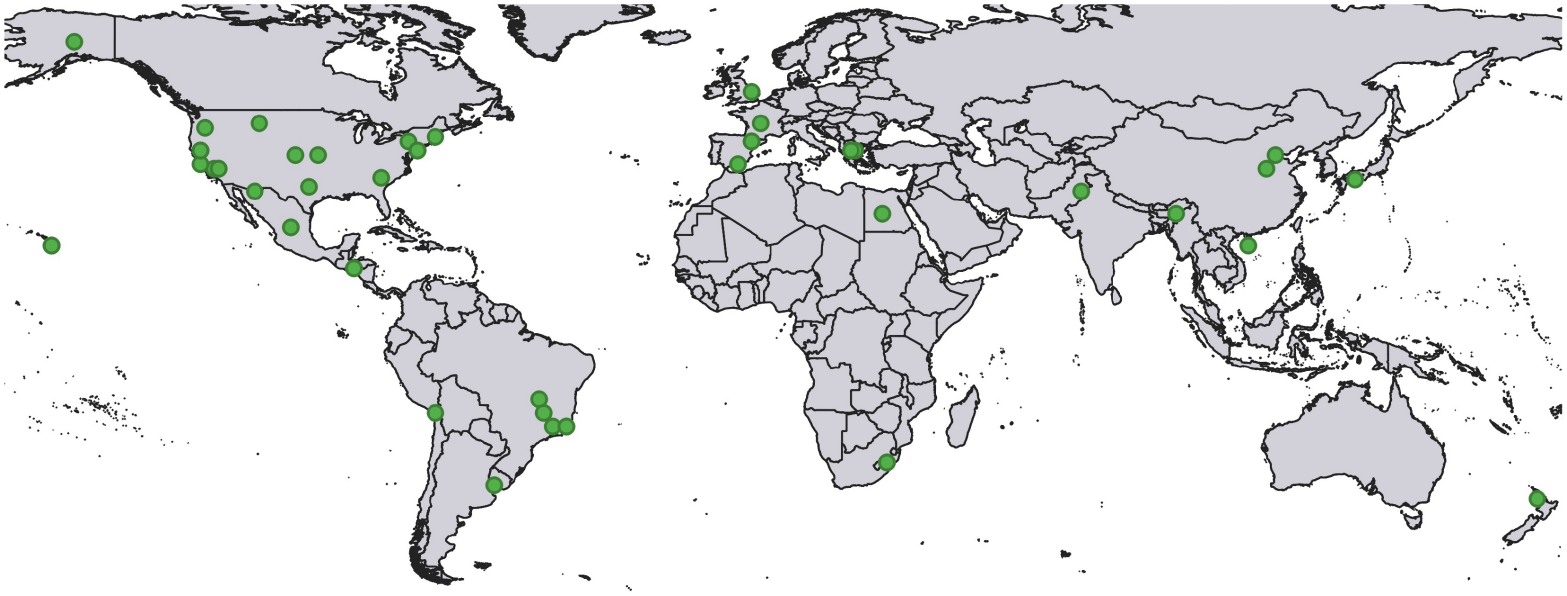
Locations from which we obtained data on life‐history processes of Neuroptera. Locations show where experiments were conducted under natural conditions or the places from which Neuroptera were collected for in vivo experiments.

A considerable number of studies on life‐history processes of Neuroptera did not include numerical data, rendering them unsuitable for our database. We retrieved 700 articles based on our search terms. From these articles, we found a total 46 studies that fit our study criteria (7% of all retrieved articles), and the data for our analyses were extracted from these 46 studies (see Supporting Material S1 for access to all studies). Of the 5937 recognized species, including 469 fossilized species of Neuroptera in the world (Zhang, 2013), our data life‐history information came from 62 species distributed in 39 geographical locations (Figure 1). Most of the data (90% of studies) originated from laboratory experiments. Peer‐reviewed literature made up the majority of our database (32 of the 46 studies). The remaining data came from 10 articles in regional journals, three book chapters, and one monograph. Most of the literature in our database was written in English (42 studies), with three articles in French and the monograph in German.

Neuroptera development was assessed within a temperature range of 9.5°C–15.0°C (5.3% of studies), 15.1°C–20.0°C (9.9% of studies), 20.1°C–25.0°C (52.5% of all studies), 25.1°C–30.0°C (9.9% of all studies), and 30.1°C–35.0°C (1.8% of all studies). The average sexual reproduction rate (the average number of offspring per year) was obtained in 29.3% of studies. Percent survival in the egg stage and during the development of all pupal stage were recorded in 9.8% and 44.9% of studies, respectively. The thermal constant was calculated in only 6.7% of cases. The photoperiod was indicated in 61.8% of studies, and humidity in 29.0% of studies. Information regarding the impact of food quantity on the development of Neuroptera was provided in only two laboratory experiments.

**FIGURE 2:**
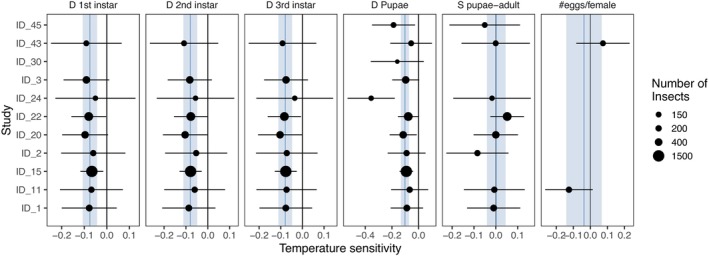
Meta‐analysis results forest plot of the temperature sensitivity for each Neuroptera study (ID). Points give the linear regression coefficient (on the linear predictor scale) between temperature and life‐history for each study, which we term temperature sensitivity. Error bars give meta‐analysis confidence limits for the temperature sensitivity for each study, and the size of the points indicates the total number of insects used in each study to calculate life‐history traits. Blue lines and shaded confidence limits give the overall pooled‐effects from meta‐analysis models. Studies are: 1—Tauber et al., 1992; 2—Tauber et al., 2006; 3‐Tauber et al., 1990; 11—Pappas et al., 2013; 15—Aghdam & Nemati, 2020; 20—Albuquerque et al., 1994; 22—Silva et al., 2006; 24—Mantoanelli et al., 2006; 30—Syrett & Penman, 1981; 43—Pappas & Koveos, 2011; and 45—Mahzoum et al., 2020.

In terms of life‐history processes, developmental times across the instar and pupal stages were most consistently recorded across different studies (58.8% of all studies). The range of duration for these periods was from 0 to 42 days for the first stage, from 0 to 81 days for the second stage, from 0 to 221 days for the third stage, and 0 to 69 for pupae (including cocoon). Fewer studies (30.1%) recorded the survival of Neuroptera after all larval, pupal, and cocoon stages, and the number of eggs per female, ranging from 0 to 1264. Only 15.0% of studies provided joint information on developmental times, survival, and reproduction. Of the 46 studies in total, we found 11 studies that had at least three repeated observations across different temperature treatments for each life‐history trait, which were used in meta‐analysis (Figure 2). We also used information from 11 studies for the multivariate analyses, although not the same selection as for the meta‐analysis (see Supporting Material S3), as here we required studies that quantified development rates, survival, and reproduction simultaneously. Classic life‐history traits, such as lifespan and generation time were recorded in fewer publications (see Supporting Material S1 for all life‐history processes our review captured). Almost all the data on the duration of the development of preimaginal stages and their temperature influence pertain to representatives of the Chrysopidae and Hemerobiidae families. Species from these families are of particular interest to scientists due to their potential as a biological method of pest control.

### 3.2 Meta‐analysis of temperature sensitivities across the life cycle

Across life cycle stages, developmental times decreased significantly with temperature (Figure 2, Supporting Material S2). For developmental times across instars 1–3, we found consistent negative impacts of temperature on life‐history traits. Pooled average temperature sensitives for developmental times of instars 1–3 were −0.075 [−0.107, −0.043; 95% Confidence limits], −0.080 [−0.111, −0.048], and −0.079 [−0.111; −0.047] (Figure 2; Figure S2.1), respectively, indicating a reduction of developmental time of approximately 8% for increase of 1°C. We also found that pupae developmental time was negatively impacted by temperature, with a pooled average sensitivity of −0.101 [−0.132, −0.071]. Conversely, survival to adult and number of eggs per female showed no clear responses to temperature, with pooled averages of 0.002 [−0.041; 0.045] and −0.037 [−0.141; 0.067], respectively, in meta‐analysis. For some species, survival was highest at an optimal temperature of 24°C–27°C, and decreased at lower and higher temperatures, but this curvilinear effect was highly uncertain (see Supporting Material S3). Furthermore, we did not find significant heterogeneity in temperature effects retained life‐history traits in meta‐analysis (Cochran's Q *p* > .05 for all traits), suggesting consistent patterns across studies.

### 3.3 Comparative analysis of lacewing life‐history strategies

The multivariate GLMMs modeling covariation in six life‐history processes mirrored the results from the meta‐analysis, showing that developmental times jointly decreased with temperature across all modeled species (Table 1, Figure S3.1), while the effect of temperature on survival from pupae to adult and number of eggs per female was highly uncertain, showing a slightly nonlinear pattern (Table 1; Figure S3.1). Latitude did not affect the covariation in life‐history processes (Table 1).

**TABLE 1:** Parameter estimates of the multivariate GLMM, modeling the covariation in six life‐history processes as a function of temperature and latitude.

Life‐history process	Mean effect (at temp = 0)	Temperature slope	Temperature^2^ slope	Latitude slope
D first instar	1.46 [1.18, 1.73]	−0.28 [−0.37, −0.20]	0.05 [0.02, 0.08]	0.03 [−0.23, 0.31]
D second instar	−0.08 [−0.28, 0.11]	−0.24 [−0.33, −0.15]	0.09 [0.05, 0.13]	0.03 [−0.36, 0.43]
D third instar	0.11 [−0.13, 0.35]	−0.27 [−0.37, −0.17]	0.08 [0.04, 0.13]	0.8 [−0.32, 0.51]
D pupae	0.92 [0.52, 1.32]	−0.21 [−0.30, −0.14]	−0.01 [−0.02, 0.07]	−0.07 [−0.39, 0.26]
# eggs/female	4.24 [3.50, 5.00]	−0.26 [−0.44, −0.06]	−0.32 [−0.43, −0.21]	0.34 [−0.21, 0.87]
S pupae‐adult	−0.41 [−0.80, −0.02]	−0.04 [−0.12, 0.04]	−0.05[−0.08, −0.03]	−0.07 [−0.24, 0.08]

*Note*: Parameters show mean values and 95% credible intervals in brackets. The model was parameterized using contrasts, so that the intercept represents the developmental times (D) of the first instar and the subsequent terms represent differences from the intercept. S—survival from pupae to adult.

**FIGURE 3:**
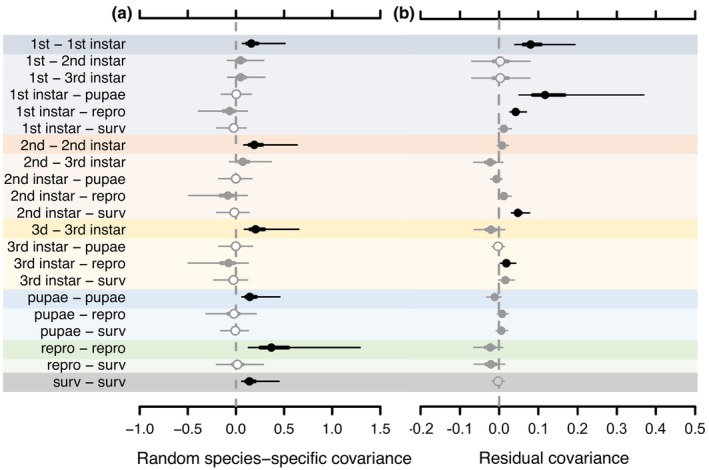
Caterpillar plots of the distribution of posterior parameters from the Bayesian multivariate mixed effect model (MCMCglmm) describing the covariance of life‐history processes in Neuroptera due to random among‐species effect on model mean and residual error. Life‐history processes include: Developmental times of first, second, and third instar and pupae stages; # eggs per female (repro); and survival rates (surv) from pupae to adult. Points represent posterior medians. Parameters where 50% credible intervals (C.I.) overlap 0 are indicated by open circles. Parameters where 50% C.I. do not but 95% C.I. do overlap 0 are indicated by closed gray circles. Parameters where 95% C.I. do overlap 0 are indicated by closed black circles. Thick lines represent 50% C.I.; thin lines represent 95% credible intervals.

A substantial amount of covariation in life‐history processes not explained by species‐specific temperature responses was captured by mean differences among species, as the random species effect on mean covariation in our models was relatively large in magnitude compared to the residual covariation (Figure 3).

**FIGURE 4:**
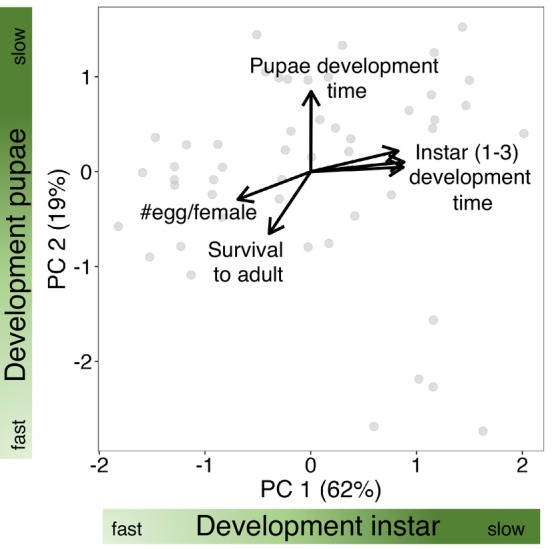
Life histories of study species' populations (points) are characterized by life‐history processes representing developmental times in different life cycle stages. To characterize life histories, a PCA was performed on the residual variation of GLMMs modeling six life‐history processes: development (in days) in three instar stages, development in pupae stage, survival from pupae to adult, and number of eggs produced per female. Arrow lengths are proportional to the loadings of each process onto the two axes.

The PCA analysis revealed that the residual covariation among the life‐history processes was adequately captured by two main PCA axes that together explained 81% of variation in life‐history processes (Figure 4). PCA axis 1 largely captured variation in instar development that traded off with survival to adult, with positive loading of developmental times in the three instar stages and negative loading of survival to adult and number of eggs per females. PCA axis 2 largely captured variation in pupae development (negative loadings) that traded off mostly with the number of eggs produced (see Table S3.1 for loadings).

Additional multivariate GLMMs modeling covariation in developmental times only (which allowed us to include a larger number of records) showed the same patterns as models based on six life‐history processes. Developmental times jointly decreased with temperature across species (Table 2; Figure S3.2), and latitude did not have an effect on developmental times, with 95% credible intervals of estimated effects overlapping 0 (Table 2). PCA analysis based on residual covariation among developmental times confirmed results from models including survival/reproduction, in addition to developmental times (Figure S3.4). Residual covariation in developmental times was adequately captured by two main PCA axes that together explained 88% of variation in life‐history processes, with PCA axis 1 largely capturing variation in instar development, while PCA axis 2 capturing variation in pupae development (see Table S3.2 for loadings).

**TABLE 2:** Parameter estimates of the multivariate GLMM, modeling the covariation in developmental times as a function of temperature and latitude.

Life‐history process	Mean effect (at temp = 0)	Temperature slope	Temperature^2^ slope	Latitude slope
D first instar	1.46 [1.15, 1.78]	−0.30 [−0.49, −0.11]	0.05 [0.02, 0.08]	0.00 [−0.00, 0.01]
D second instar	−0.12 [−0.38, 0.11]	−0.26 [−0.47, −0.06]	0.07 [0.01, 0.12]	0.01 [−0.00, 0.01]
D third instar	0.11 [−0.27, 0.49]	−0.28 [−0.53, −0.04]	0.01 [−0.07, 0.10]	0.01 [0.00, 0.02]
D pupae	1.15 [0.86, 1.43]	−0.25 [−0.44, −0.06]	0.07 [0.05, 0.10]	−0.01 [−0.01, 0.01]

*Note*: Parameters show mean values and 95% credible intervals in brackets. The model was parameterized using contrasts, so that the intercept represents the developmental times (D) of the first instar and the subsequent terms represent differences from the intercept. S—survival from pupae to adult.

Lastly, multivariate GLMMs modeling covariation in survival and reproduction (and thus including a larger sample size than when modeling six life‐history processes) confirmed that both life‐history processes were weakly affected by temperature, with only # eggs/female showing a significantly negative response to temperature (Table 3; Figure S3.5). Neither latitude nor study type had an effect on survival or reproduction, with 95% credible intervals of estimated effects overlapping 0 (Table 3).

**TABLE 3:** Parameter estimates of the multivariate GLMM, modeling the covariation in survival (S) and reproduction (# eggs/female) as a function of temperature, latitude, and literature source.

Life‐history process	Mean effect (at temp = 0)	Temperature slope	Temperature^2^ slope	Latitude slope	Literature source
Intercept (S pupae‐adult)	1.07 [0.95, 1.19]	−0.04 [−0.16, 0.07]	−0.07 [−0.10, −0.04]	−0.07 [−0.21, 0.06]	0.15 [−0.21, 0.50]
#eggs/female	4.69 [4.31, 5.11]	−0.24 [−0.43, −0.04]	−0.39 [−0.463, −0.28]	0.24 [−0.25, 0.71]	−0.85 [−2.37, 0.65]

*Note*: Parameters show mean values and 95% credible intervals in brackets. The model was parameterized using contrasts, so that the intercept represents the survival from pupae to adult and the subsequent terms represent differences from the intercept. S—survival from pupae to adult.

## 4 DISCUSSION

Our research aimed to consolidate information regarding the simultaneous influence of temperature on various stages of development of lacewings across species. The ambient temperature represents a pivotal abiotic factor for insects that lack the ability to maintain a constant body temperature. Insects consistently grapple with temperature variation to evade the detrimental consequences of heat stress. In order to mitigate these risks, insects have evolved an array of physiological and behavioral thermoregulation mechanisms, along with molecular responses that facilitate their survival and function across diverse temperature conditions. Elevated temperature sensitivity exerts an influence on the development and growth of insects and may result in phenological alterations (Chmura et al., 2018; Kong et al., 2019; Rebaudo & Rabhi, 2018). Evaluating and quantifying the temperature sensitivity of insect life‐history processes throughout the life cycle, will allow us to better understand and forecast their phenological reactions to climatic stress (Jasrotia et al., 2022; von Schmalensee et al., 2021).

Our results clearly show that temperature significantly influences the duration of Neuroptera development. Within the viable temperature range, increasing temperatures are associated with an acceleration of growth and development in Neuroptera, along with increased metabolic activity, facilitating faster development of eggs, larvae, and pupae (Pappas et al., 2013; Ranjbar & Nemati, 2020; Syrett & Penman, 1981; Tauber & Tauber, 2015). Our results indicate that such faster development is not necessarily associated with lower survival and reproduction, something that has been described for other insects as well (Ju et al., 2015). However, this may only be the case under certain temperature conditions (Abarca et al., 2024). Our results are in line with previous studies suggesting that high and low temperatures can substantially decrease survival and reproductive output and thus population viability (Aghdam & Nemati, 2020; Mantoanelli et al., 2006; Pappas et al., 2013). For instance, if individuals develop faster and have relatively smaller body sizes, high temperatures can then induce further stress and lead to relatively more moisture loss, increased evaporation, and overheating in females, which clearly has a negative impact on survival and reproductive function (Aghdam & Nemati, 2020; Albuquerque et al., 1994). Similar responses of development versus survival in responses to temperature have been found in other insect species, for instance, *Platyptilia carduidactyla* (Kingsolver & Buckley, 2020).

The lack of correlation, as seen in our PCA analysis, between the development at the pupal stage and the development at the instar stages in Neuroptera suggest the presence of life‐history trade‐offs at the species level. Although we cannot discard other mechanisms, for instance, unobserved phenotypic, behavioral, or environmental differences among the compared species, knowledge on the thermal biology of Neuroptera supports the presence of life‐history trade‐offs resulting from ecological and evolutionary adaptations that affect the different stages of their life cycle. Environmental conditions can vary significantly between the larval stage and the pupal and cocoon stages. Neuroptera larvae have an active metabolism and are predators adapted to specific environmental conditions where they live and hunt (Bolok et al., 2010; Botti et al., 2022; Boyden, 1983; Breene et al., 1992). All stages may undergo similar influences regarding food selection and predation. These stages involve similar physiological processes designed for growth and development. Meanwhile, the pupal and cocoon stages are subject to other influences, such as the impact of abiotic factors on metabolism and development. These stages usually do not interact with the external environment and may be less adapted to external conditions (Aspöck et al., 1980). These stages are also highly metabolically active in insects (Raglan et al., 2009), as individuals expend energy on the transformation from the larval state to the adult insect (metamorphosis), involving restructuring and degradation of certain larval tissues (Zhao et al., 2020, 2022).

Our comparative analyses also confirmed previous studies supporting the idea that early and reproductive stages are more sensitive to extremely high temperatures compared to the mature larval stage (Ma et al., 2021; Zhao et al., 2017). For instance, Ma et al. (2021) suggest that the varying impact of extremely high temperatures might be due to the different thermal environments inhabited and adapted to by various stages (Kingsolver et al., 2011; Ma et al., 2021; Pincebourde & Casas, 2015). Furthermore, body size may also explain why larger larvae are more heat‐tolerant than smaller early life stages (Kingsolver et al., 2011; Ma et al., 2021; Zhao et al., 2017), as smaller larvae are more prone to rapid increases in body temperature and water loss. At the same time, our results contradict the hypothesis that immobile stages of insects (eggs and pupae) should be more resistant to heat compared to mobile adult stages and larval stages due to their lower ability for behavioral thermoregulation (Huey et al., 2003). We found that across species, the pupal stage of Neuroptera is also sensitive to high temperatures. We believe that at this stage, the insect is unable to regulate its body temperature through mechanical means, unlike a mobile larva that can seek shade. Most Neuroptera species seek shaded places, such as cracks in tree bark, leaf litter, and other hidden locations, for pupation (Canard & Volkovich, 2001). Under optimal conditions, as demonstrated in the study by Canard & Principi in 1984, *C. regalis* only experiences a minimal weight loss due to dehydration and respiration during the pupal stage, less than 7% of its initial weight by the end of diapause (Canard & Principi, 1984). However, there are few studies on the impact of extreme temperatures during the pupal stage, and we are unaware of the potential harm caused by overheating and significant water loss at this stage.

Our work also points to important knowledge gaps. We found no studies that assessed the survival of adults under temperature variation. Adults can show a high variation in feeding strategies, and the success of these strategies can be strongly affected by temperature responses during previous developmental stages (Devetak & Klokočovnik, 2016). Studies are also lacking on how physiological responses of individuals during diapause mediate the effects of temperature extremes on other life‐history processes, and vice versa. This is despite the fact that understanding joint effects of temperature variation across seasonally varying life cycle processes is critical (Williams et al., 2015). For instance, a recent study has shown that *Chrysoperla pallida* can display high phenotypic plasticity in metabolic scaling under high temperatures, with a trade‐off between body size and mandible size, where larger individuals developed smaller mandibles, which subsequently improved survival during diapause (Álvarez & Ruano, 2024). In addition, few studies assessed the effects of various environmental drivers on life‐history processes although it is known that local habitat conditions may mask effects of climate (Musolin & Saulich, 2012; Pollard et al., 1995; Roy et al., 2001).

Neuroptera provide an excellent example of how our understanding of the mechanisms that mediate climate‐induced changes in invertebrate life‐history dynamics is limited. While each species in this order has a critical temperature maximum, they also possess apparent mechanisms to mitigate adverse effects. For instance, many Neuroptera species are active during twilight or at night, cover themselves with chitin remnants after molting, use plant fragments for camouflage, and seek shaded habitats. Larvae of Myrmeleontidae can burrow deeper into the sand or move to shaded areas when the upper layer becomes excessively heated. All these behavioral adaptations reduce the risk of overheating. Additionally, many Neuroptera species exhibit adaptive abilities regarding diapause duration and survival (Álvarez & Ruano, 2024). Lastly, all Neuroptera larvae and the majority of species are active predators, and their population size correlates with that of their prey. This includes the dynamics of correlations influenced by seasonality or phenological adaptations of Aphidoidea with Chysopidae, Hemerobiidae, Coniopterygidae, or Formicidae with Myrmeleontidae. In our review, no single study assessed how such complexities affect life‐history processes across the life cycle. A comprehensive approach understanding these factors is required to predict population dynamics and the consequences of climate change.

Despite remaining knowledge gaps, our results on the variation of life‐history processes in Neuroptera and how much of this variation is explained by temperature paints a nuanced picture of potential population dynamics under climate change. They demonstrate that predicting responses to future thermal conditions requires a mechanistic understanding of how organisms react to a wide range of temperatures experienced throughout their life cycle, not just at specific phases of the life cycle (Sinclair et al., 2016). Faster developmental times may not necessarily lead to higher abundances due to differential temperature responses across the life cycle. Such buffering effects across the life cycle are commonly investigated in mammals and birds (Jackson, Johnson, et al., 2022; Jackson, Le Coeur, & Jones, 2022; Paniw et al., 2018) and provide an exciting new area of research for insects (Lackey et al., 2023), which cover different ecological and evolutionary niches and are thus governed by different physiological trade‐offs and adaptations.

## AUTHOR CONTRIBUTIONS

**Hanna Serediuk:** Conceptualization (equal); data curation (lead); investigation (equal); validation (lead); writing – original draft (equal); writing – review and editing (supporting). **John Jackson:** Formal analysis (equal); methodology (equal); writing – review and editing (supporting). **Sanne Maria Evers:** Conceptualization (supporting); formal analysis (supporting); validation (equal); writing – review and editing (equal). **Maria Paniw:** Conceptualization (lead); formal analysis (equal); funding acquisition (lead); methodology (equal); supervision (lead); writing – original draft (lead); writing – review and editing (lead).

## FUNDING INFORMATION

This work was funded by the Consejo Superior de Investigaciones Científicas (CSIC) with the grant UCRAN20052. MP was additionally funded by the grant RYC2021‐033192‐I by MCIN/AEI/10.13039/501100011033 by “European Union NextGenerationEU/PRTR.”

## CONFLICT OF INTEREST STATEMENT

We declare no conflict of interest.

### OPEN RESEARCH BADGES

This article has earned Open Data and Open Materials badges. Data and materials are available at [[insert provided URL(s) on the Open Research Disclosure Form]].

## Supporting information


Supplementary Material S1.

Supplementary Material S2.

Supplementary Material S3.

Data S1.

## Data Availability

All data and R scripts to run the comparative analyses can be found on GitHub and will be deposited on Zenodo upon acceptance of the manuscript: https://github.com/MariaPaniw/lacewings_life_histories. Literature that we extracted for the review can be found here: https://drive.google.com/drive/folders/1jWptakO8ea5g_97oe0XaUMgc4MzH4rsK?usp=sharing.
