# Supplementary material for: Comparative life‐history responses of lacewings to changes in temperature
Source: Ecol Evol. 2024 Jul 18;14(7):e70000. doi: 10.1002/ece3.70000 (PMC11257770; doi:10.1002/ece3.70000)
Supplement: Supplementary file 1 — Supplementary Material S1. [file ECE3-14-e70000-s001.pdf]

Supporting Material S1 – Review protocol and data extracted for

Comparative life-history responses of lacewings to changes in temperature

Literature that we extracted for the review can be found here:

https://drive.google.com/drive/folders/1jWptakO8ea5g_97oe0XaUMgc4MzH4rsK?usp=sharing

Detailed Extraction Protocol and Data Description

For each Neuroptera species we searched Web of Science and Scopus for literature for studies (published before March 31, 2023) that quantified the effects of temperature on different life-history processes, such as the rate of development, lifespan, and reproductive activity. Additionally, we employed Google Scholar to locate articles that may not have been indexed in the aforementioned databases. We used the following search terms:

"(Neuroptera AND life cycle)" OR "(Chrysopidae AND life cycle)" OR "(Hemerobiidae AND life cycle)" OR "(Myrmeleontidae AND life cycle)" OR "(Coniopterigydae AND life cycle)" OR "(Sisyridae AND life cycle)" OR "(Ascaphidae AND life cycle)" "(Dilaridae AND life cycle)" OR "(Polystoechotidae AND life cycle)" OR "(Psychopsidae AND life cycle)" OR "(Coniopterygidae AND life cycle)" OR "(Ithonioidea AND life cycle)" OR "(Berothidae AND life cycle)" OR "(Mantispidae AND life cycle)" OR "(Nemopteridae AND life cycle)" OR "(Ascalaphidae AND life cycle)" OR "(Nemopteridae AND life cycle)" OR "(Nymphidae AND life cycle)" OR "(Osmylidae AND life cycle)" OR "(Nevrorthidae AND life cycle)" OR "(Sisyridae AND life cycle)" OR "(Neuroptera life cycle AND temperature)" OR "(Temperature AND Neuroptera development)" OR "(Temperature AND Chrysopidae development)" OR "(Temperature AND Hemerobiidae development)" OR "(Temperature AND Neuroptera life history)" OR "(Neuroptera life history)" OR "(Neuroptera temperature adaptation)" OR "(Climate change AND Neuroptera)" OR "(Climate change impact AND insects)".

From any study that met our search terms, we extracted information on the relationship between temperature and life cycle only when the latter was quantitatively associated with temperature variation, i.e., we did not extract information in the following cases:

- The research was descriptive and did not provide numerical evidence for variation in life-history processes under temperature variation.
- The research was based on obtaining information about a specific stage of the life cycle, for example, the study of diapause (such as in Li et al., 2018).

- The research was based on the analysis of the influence of other factors, and temperature was not taken into account, for example, the study of the effect of different photoperiods on Neuroptera (Yadav et al., 2008).
- The research described the development characteristics of species (Viana & Albuquerque, 2009) or the behavioral traits of different stages (for example, Tauber et al., 2014).
- The fertility of females was studied under the influence of prolonged storage of pupae in a diapause state (Chang et al., 2000) or the description of the mass rearing process without specifying the cultivation conditions (temperature, humidity, etc.) and without indicating the development time of each stage (Ruzicka, 2010).

We note that we may miss a potentially relevant study if the search terms were not mentioned in the title, abstract or keywords.

Description of key collected data

From all studies quantifying the relationship between temperature and the duration of development of stages of the life cycle of Neuroptera, we obtained the following information:

- Geographical location – the center of the studied territory was always used. If coordinates were not specified in the study, we assigned coordinates based on descriptions of field studies and data collection.
- Habitat, if the place of the experiment and its brief description is indicated, or where the material for laboratory research was collected.
- Experimental environment — controlled environment or field conditions.
- Relationship between different temperature levels (experimental levels where appropriate) and life-history process. Raw data from experiments was not provided in any study. Instead, the information was provided as percentage success or mean response (and sometimes 9% C.I. in response) to each environmental condition explored. For key life cycle stages, we recorded

developmental time, survival rate, and reproductive rate in response to temperature.

Detailed data description

1. Study descriptors

a. St_ID

The ID of the study which links to the folder where all the primary sources for the database are located: https://drive.google.com/drive/folders/1jWptakO8ea5g_97oe0XaUMgc4MzH4rsK?usp=sharing

2. Location data

a. Latitude and longitude

The latitude and longitude of the specific study site (as specified in the manuscript) were recorded in decimal degrees using the WGS84 global projection. If latitude and longitude were not reported in the original manuscript, we used a verbal description of the study location (e.g., the nearest city, the center of the national park, etc., where the study was conducted) to estimate these values. This approximation of the study site did not affect our analysis and conclusions.

b. Habitat

If the source we used provided information about the habitat, we recorded this data in our database. Neuroptera were most commonly collected in gardens, including citrus, olive, peach, pear, and mixed fruit gardens. Additionally, for one of the experiments, insects were gathered in an agroecosystem in cotton fields. Furthermore, specific collection locations were indicated, such as coniferous and deciduous forests (*Quercetum ilicis*).

2. Experimental Conditions

We included studies conducted under field conditions (**in situ**) as well as laboratory experiments (**in vivo**). We also recorded the average number of individuals used per treatment (**Instances_count**)

3. Temperature

a. Constant Temperature

We recorded a constant temperature (t) used during the research in degrees Celsius (°C).

b. Thermal Constant

If available, we also indicated the thermal constant "K," representing the heat requirement for development and measured in degree-days (calculated using the equation K = (1/y) (x - t), where y = the average development rate, and x = temperature (°C)).

c. *Minimum, Optimal, and Maximum Temperatures*

In one of the studies, we documented the minimum, optimal, and maximum temperature treatments for several pre-imaginal stages. In our database, we marked the following: **tmin_E** - minimum temperature required for egg development; **topt_E** - optimal temperature for egg development; **tmax_E** - maximum temperature at which egg development occurs; **tmin_1st_L** - minimum temperature required for the development of first-stage larvae; **topt_1st_L** - optimal temperature for the development of first-stage larvae; **tmax_1st_L** - maximum temperature at which first-stage larvae develop; **tmin_2nd_L** - minimum temperature required for the development of second-stage larvae; **topt_2nd_L** - optimal temperature for the development of second-stage larvae; **tmax_2nd_L** - maximum temperature at which second-stage larvae develop; **tmin_3rd_L** - minimum temperature required for the development of third-stage larvae; **topt_3rd_L** - optimal temperature for the development of third-stage larvae; **tmax_3rd_L** - maximum temperature at which third-stage larvae develop; **tmin_P&P** - minimum temperature required for the development of pupae and prepupae; **topt_P&P** - optimal temperature for pupae and prepupae development; **tmax_P&P** - maximum temperature at which pupae and prepupae develop.

4. Other Influencing Factors on Neuroptera

a. Photoperiod

If the experiments provided information about the ratio of light to dark hours (photoperiod, **P (hours)**), we recorded this information in our database.

b. Humidity

If the experiment specified constant environmental humidity conditions, we indicated the humidity percentage (**Humid. (%)**).

c. Feeding

In some experiments, the feeding regimen and the quantity of food consumed by the Neuroptera larvae were specified. We recorded the number of prey used to feed the Neuroptera larvae daily

(**Food_(pieces/day)**).

5. Duration of Different Life Cycle Periods

We recorded the duration in days of various life cycle periods of Neuroptera if this information was available in the articles. In our database, these durations are denoted as follows: **Preovi_per (d/M) -** duration of the pre-reproductive period of females in days (average); **Ovi_rate (d/M) -** duration of the reproductive period of females in days (average); **Postovi_per (d/M) -** duration of the post-reproductive period of females in days (average); **E_dev (d/M) -** duration of egg development in days (average); **Dev_1st_inst (d/M) -** duration of the first-stage larval development in days (average); **Dev_2nd_inst (d/M) -** duration of the second-stage larval development in days (average); **Dev_3rd_inst (d/M) -** duration of the third-stage larval development in days (average); **Dev_P&P (d/M) -** duration of pupal and prepupal development in days (average); **Lif_exp_post_mat (d/min) -** minimum expected post-maturity lifespan in days; **Lif_exp_post_mat (d/max) -** maximum expected post-maturity lifespan in days; **Lif_exp_post_mat (d/M) -** average expected post-maturity lifespan in days.

6. Demographic Indicators

The studies contained in the dataset quantitatively determined demographic indicators in various ways. Consequently, we grouped the indicators presented in each article as related to survival, reproductive success, reproductive productivity, growth/development, population status, or population growth. Here, we describe how we assigned attributes from individual studies to each of these classes.

Survival **-** In our database, we represented indicators of egg-laying survival as **E_surv (%)** and larval survival as **L_surv (%)**.

Reproductive Success and Productivity **-** Studies with quantitative assessments of reproduction could record the percentage of egg-laying females in one generation as **Ovip_fem (%)** and the percentage of fertile females in one generation as **Fert_fem (%).** We recorded female fecundity in the database through reproductive indicators, namely, how many females in a new generation emerge from a single

female (**Repr_rate (f/f)**), and the number of eggs laid by a single female - minimum (**M_sex_rep_rate (1f/min)**), maximum (**M_sex_rep_rate (1f/max)**), and average (**M_sex_rep_rate (1f/M)**). We also noted the male-to-female ratio in the generation (**Pr_sex_rat**) among those born in a particular generation. Additionally, we indicated the minimum (**N_gen (min)**) and maximum (**N_gen (max)**) possible number of generations within one year.

Variables that quantitatively determined generation time, intrinsic rate of natural increase (days-1), and generation doubling time were included as growth indicators. We specified the minimum (**Gen_tim (min)**), maximum (**Gen_tim (max)**), and average (**Gen_tim (M)**) generation time. The intrinsic rate of natural increase (**Intr_rate_nat_incr (days-1)**) measures the population's level of reproduction and mortality. Generation doubling time (**Doubl_tim**) is the number of days required for a population to double its size.

Summary of results

The majority of our data was sourced from peer-reviewed literature (69.5%) (32). Our analysis incorporated one monograph and three book chapters. We also included data from articles in regional journals, which accounted for 21.8% (10) of the total. Overall, more than 91% (42) of the sources used were written in English, with three articles in French and one book in German. In retrospect, the distribution of published works is as follows: from 2024 to 2015, there were six sources (13%); from 2014 to 2005, there were 18 sources (39.1%); from 2004 to 1995, there were four sources (8.7%); from 1994 to 1985, there were 11 sources (23.9%); from 1984 to 1975, there were four sources (8.7%); from 1974 to 1965, there were two sources (4.4%); and one publication was from 1992 (2.2%).

Additional references

Chang, Y.-F., Tauber, M. J., Tauber, C. A., & Nyrop, J. P. 2000. Interpopulation variation in Chrysoperla carnea reproduction: implications for mass-rearing and storage. Entomologia Experimentalis et Applicata 95, 293–302.

Li, Y., Wang, M., Gao, F., Zhang, H., Chen, H., Wang, M., Liu, C., & Zhang, L. 2018. Exploiting

diapause and cold tolerance to enhance the use of the green lacewing Chrysopa Formosa for biological control. Biological Control 127, 116–126.

Ruzicka, Z. 2010. Detection of oviposition-deterring larval tracks in Chrysopa oculata and Chrysopa perla (Neuroptera: Chrysopidae). European Journal of Entomology 107, 65–72.

Tauber, C. A., Tauber, M. J., & Albuquerque, G. S. 2014. Debris-carrying in larval Chrysopidae: Unravelling its evolutionary history. Annals of the Entomological Society of America 107, 295–314.

Viana, G. G., & Albuquerque, G. S. 2009. Polimorfismo no padrão de manchas tegumentares de larvas e adultos de Ceraeochrysa caligata (Neuroptera: Chrysopidae) e redescrição dos instares larvais. Zoologia (Curitiba) 26, 166–174.

Yadav, A., He, X. Z., & Wang, Q. 2008. Effect of photoperiod on development and reproduction in Tasmanian lacewing Micromus tasmaniae (Walker) (Neuroptera Hemerobiidae). New Zealand Plant Protection 61, 338–342.
