# Supplementary material for: Comparative life‐history responses of lacewings to changes in temperature
Source: Ecol Evol. 2024 Jul 18;14(7):e70000. doi: 10.1002/ece3.70000 (PMC11257770; doi:10.1002/ece3.70000)
Supplement: Supplementary file 2 — Supplementary Material S2. [file ECE3-14-e70000-s002.pdf]

Supporting Material S2 – Additional results of meta-analysis

To replicate the analyses, (1) run `metaanalysis_aquisition_exploration_may24.R`, followed by (2) `metaanalysis_study_regressions_may24.R`, and then (3) `metaanalysis_analyses_may24.R`

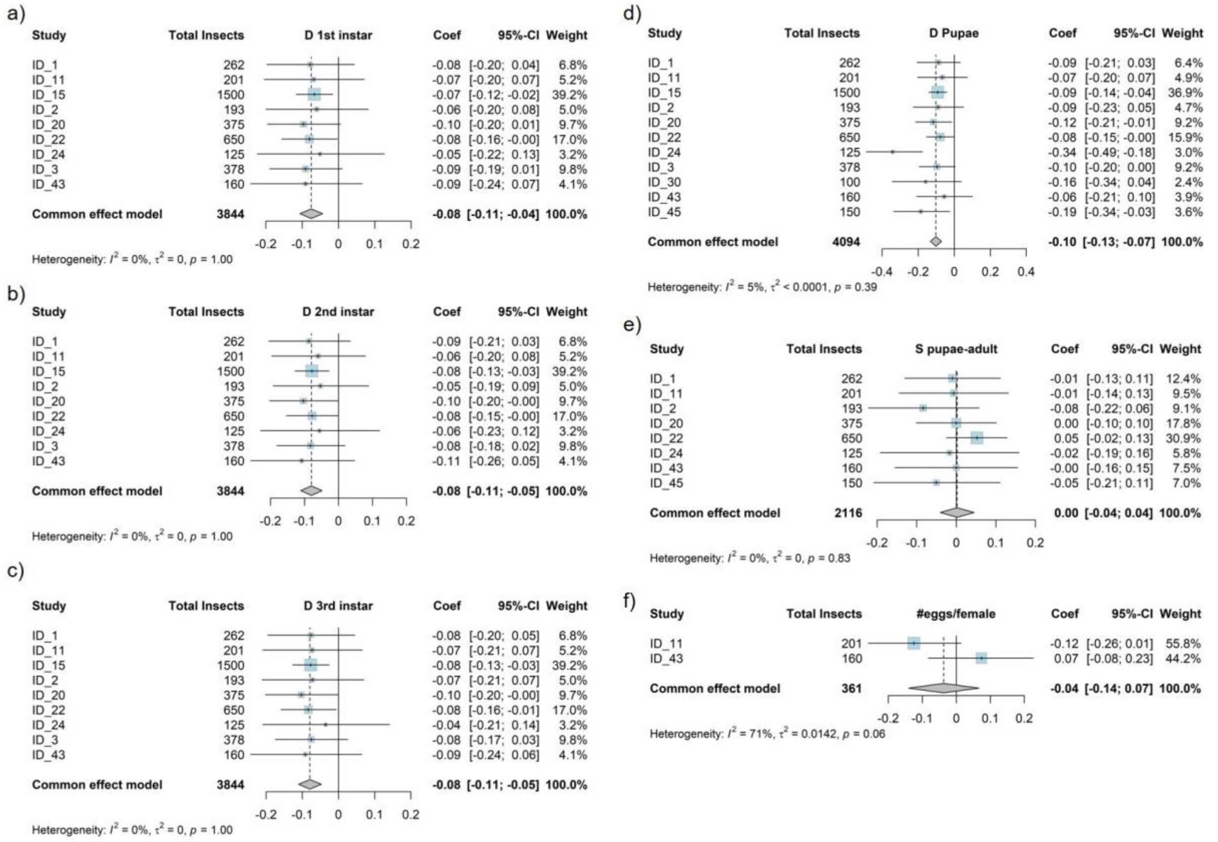

Figure S2.1. Meta-analysis results in full for the effect of temperature on life-history traits in *Neuroptera*. Forest plots for development time in the 1-3rd instar (a-c), development time for pupae (d), survival of pupae to adulthood (e), and number of eggs per female (f). In each plot, each row gives the model-derived coefficient for each study (blue box) and 95% confidence level (error bar; also presented numerically), with the total sample size (number of insects), and % weighting to the pooled effect. Common effect model gives the overall pooled effect with 95% confidence limits. Statistics for study heterogeneity are given below each forest plot, with I^2 (percentage of variation due to heterogeneity), τ^2 , and the p-value of Cochran's Q. Studies are: 1-Tauber et al., 1992; 2-Tauber et al., 2006; 3-Tauber et al., 1990; 11-Pappas et al., 2013; 15-Aghdam & Nemati, 2020; 20-Albuquerque et al., 1994; 22-Silva et al., 2006; 24-Mantoanelli et al., 2006; 30-Syrett & Penman, 1981; 43-Pappas & Koveos, 2011; 45-Mahzoum et al. 2020
