# Supplementary material for: Comparative life‐history responses of lacewings to changes in temperature
Source: Ecol Evol. 2024 Jul 18;14(7):e70000. doi: 10.1002/ece3.70000 (PMC11257770; doi:10.1002/ece3.70000)
Supplement: Supplementary file 3 — Supplementary Material S3. [file ECE3-14-e70000-s004.pdf]

Supporting Material S3 – Additional results of comparative life-history analyses

Analyses of life-history processes including 6 life-history processes (n = 51)

See `mcmc_pca_Neuroptera_main_text.R`

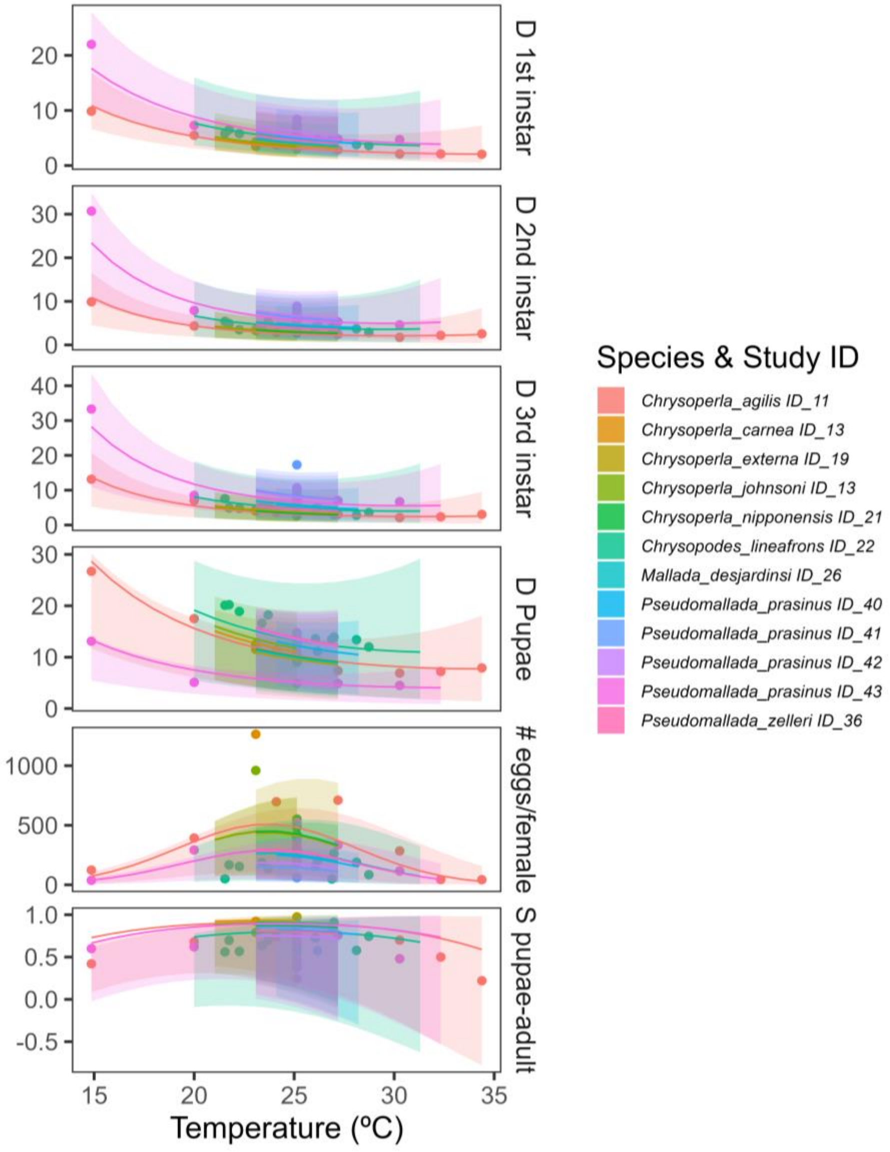

Figure S3.1. Covariation in life-history responses to temperature across 9 Neuroptera species and 11 studies (colors). Points are observed values from the literature review. Lines are species by study specific predictions of developmental (D) times (in days) for three instar and pupae stages, number of eggs per female, and proportion of pupae surviving (S) to adult stages from the multivariate MCMCglmm. colored areas are 95 % credible intervals of model predictions.

Table S3.1 Loadings of life-history processes (rows; see Fig. S1.1 for description) on the principal component axes. The first two axes, which together explain 76% of the variation in the data and have associated eigenvalues > 1 (in bold), are varimax-corrected. Redder colors identify more positive loadings while bluer colors identify more negative loadings. % var. – percent variation explained by each principal component axis; Cumulative var. – cumulative variance explained.

Life-history trait	PCA 1	PCA 2	PCA 3	PCA 4	PCA 5	PCA6
D 1st instar	0.89	0.23	0.03	-0.17	0.76	0.34
D 2nd instar	0.94	0.00	-0.15	-0.35	-0.09	-0.74
D 3rd instar	0.95	0.1	-0.05	-0.12	-0.62	0.54
D Pupae	0.00	0.94	0.07	-0.51	-0.07	0.04
S pupae-adult	-0.42	-0.71	-0.79	-0.43	0.07	0.17
#eggs/female	-0.74	-0.31	0.57	-0.61	-0.02	0.06
Eigenvalue	**1.92**	**1.06**	0.72	0.62	0.38	0.28
% Var.	61.5	18.9	8.6	7.2	2.4	1.3
Cumulative var.	61.5	80.4	89.3	96.2	98.6	100.0

Analyses of life-history processes including 4 life-history processes (n = 120)

See `mcmc_pca_Neuroptera_dev_only.R & mcmc_Neuroptera_in_situ.R`

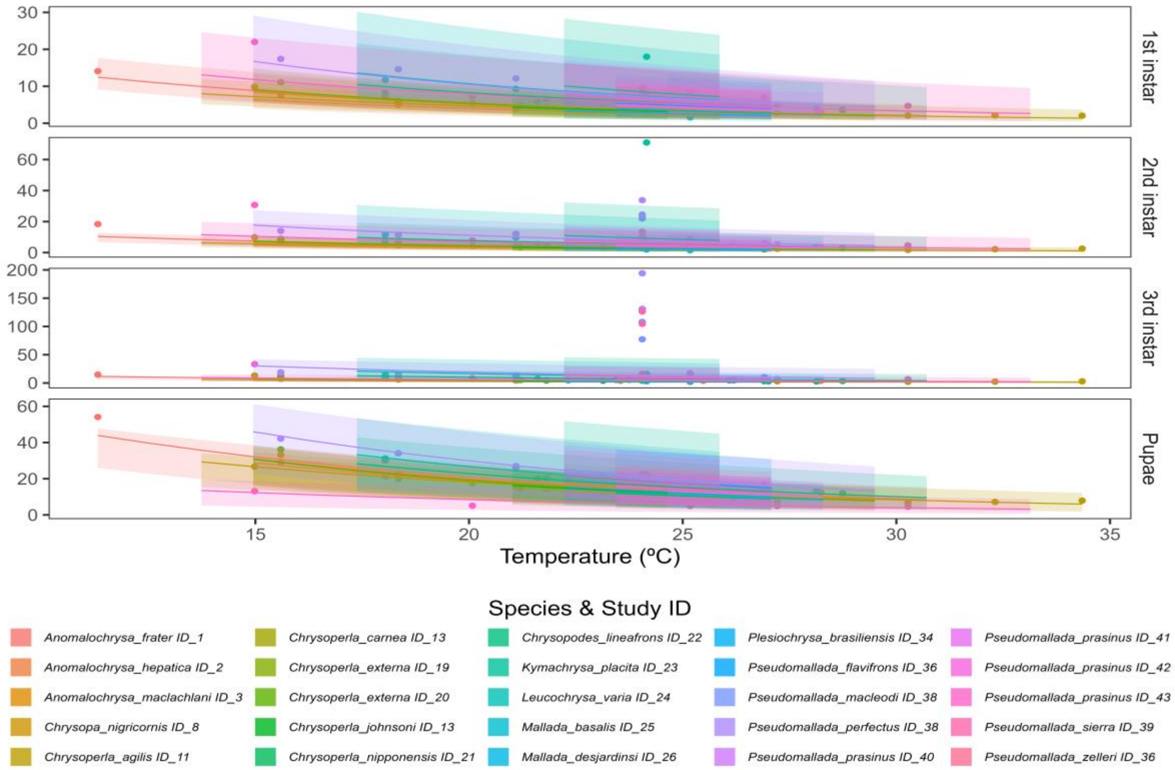

Figure S3.2. Covariation in life-history responses to temperature across 25 Neuroptera species and studies combinations (colors). Points are observed values from the literature review. Lines are species and study specific predictions of developmental times (in days) for three instar and pupae stages from the multivariate MCMCglmm. Coloured areas are 95 % credible intervals of model predictions.

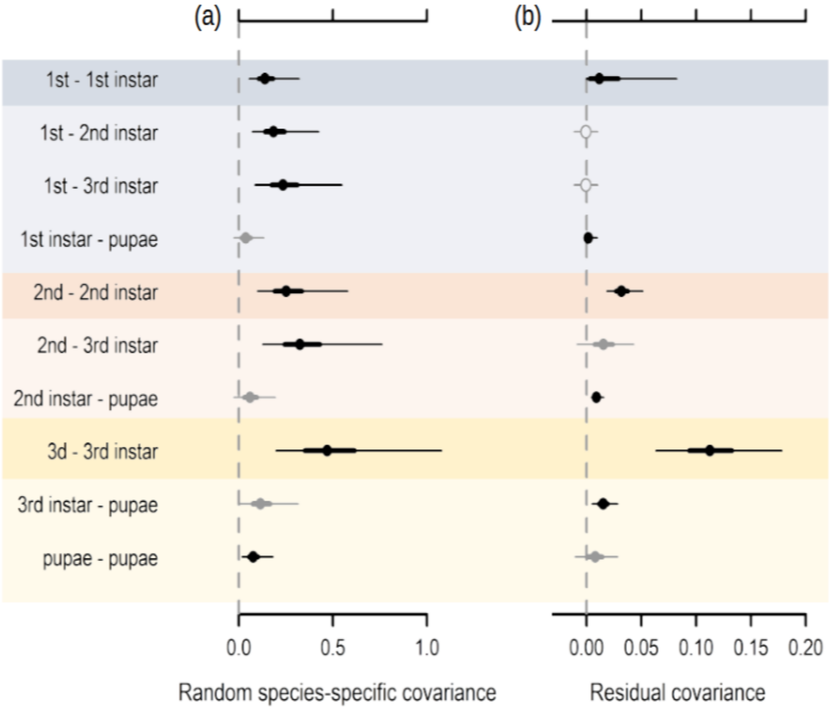

Figure S3.3. Caterpillar plots of the distribution of posterior parameters from the Bayesian multivariate mixed effect model describing the covariance of life-history processes in Neuroptera due to random among-species effect and residual error. Life-history processes include: developmental times of 1st, 2nd, and 3rd instar and pupae stages. Parameters where 50% credible intervals (C.I.) overlap 0 are indicated by open circles. Parameters where 50% C.I. do not but 95% C.I. do overlap 0 are indicated by closed gray circles. Parameters where 95% C.I. do overlap 0 are indicated by closed black circles. Thick lines represent 50% C.I.; thin lines represent 95% credible intervals.

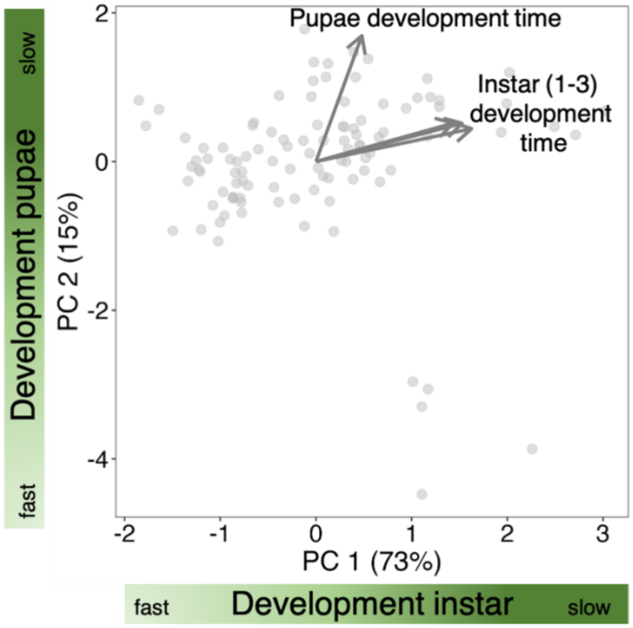

Figure S3.4. After excluding survival and reproduction, life-history strategies of study species' populations (points) remain characterized by differences in developmental times in different life cycle stages. To characterize life histories, a PCA was performed on the residual variation of GLMMs development (in days) in 3 instar stages and in the pupae stage. Arrow lengths are proportional to the loadings of each process onto the two axes.

Table S3.2 Loadings of life-history processes (rows; see Fig. S1.1 for description) on the principal component axes. The first two axes, which together explain 89% of the variation in the data and have associated eigenvalues > 1 (in bold), are varimax-corrected. Redder colors identify more positive loadings while bluer colors identify more negative loadings. % var. – percent variation explained by each principal component axis; Cumulative var. – cumulative variance explained.

Life-history trait	PCA 1	PCA 2	PCA 3	PCA 4
D 1st instar	0.52	-0.22	0.56	0.584
D 2nd instar	0.54	-0.30	0.17	-0.772
D 3rd instar	0.50	-0.18	-0.79	0.246
D Pupae	0.4	0.9	0.03	-0.036
Eigenvalue	**1.70**	0.8	0.61	0.34
% Var.	72.5	15.1	9.2	3.0
Cumulative var.	72.5	87.7	97.0	100.0

Analyses of life-history processes including 2 life-history processes (n = 56)

Here, we also included study type (either peer-reviewed or non-peer reviewed, grey literature) as a covariate.

See `mcmc_Neuroptera_surv_repro_only.R`

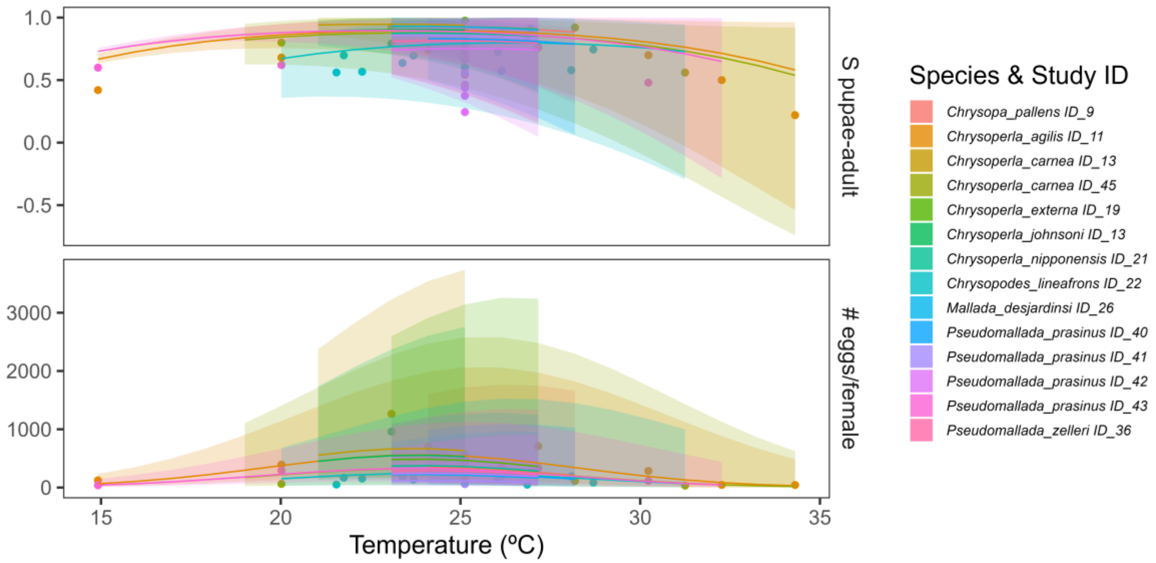

Figure S3.5. Covariation in life-history responses to temperature across 10 Neuroptera species (point colors). Points are observed values from the literature review. Lines are mean predictions of survival (S) and number of eggs per female from the multivariate MCMCglmm. Grey area are 95 % credible intervals of model predictions.

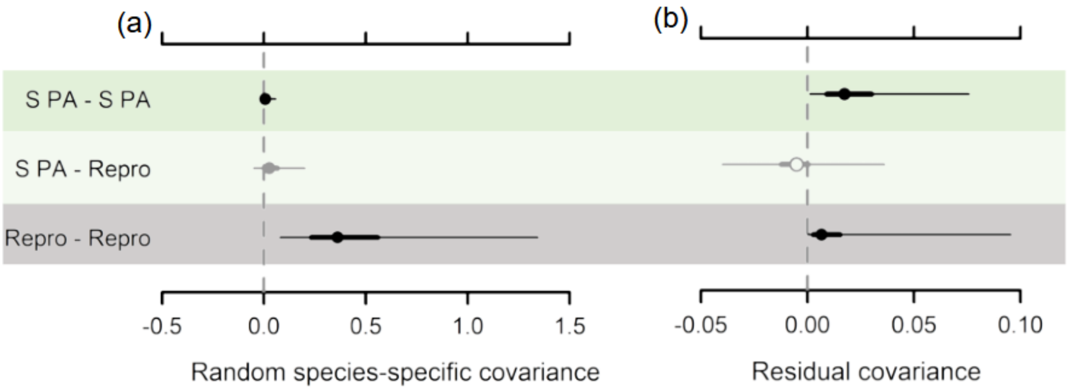

Figure S3.6. Caterpillar plots of the distribution of posterior parameters from the Bayesian multivariate mixed effect model describing the covariance of life-history processes in Neuroptera due to random among-species effect and residual (within-species) error. Life-history processes include survival (S) of pupae to adult (PA) and #eggs/female (Repro). Parameters where 50% credible intervals (C.I.) overlap 0 are indicated by open circles. Parameters where 50% C.I. do not but 95% C.I. do overlap 0 are indicated by closed gray circles. Parameters where 95% C.I. do overlap 0 are indicated by closed black circles. Thick lines represent 50% C.I.; thin lines represent 95% credible intervals.

References

Brooks, S.P., Gelman, A. 1998. General methods for monitoring convergence of iterative simulations. Journal of Computational and Graphical Statistics 7, 434–455.

Brommer, J., Class, B., Covarrubias-Pazaran, G. 2019. Multivariate mixed models in ecology and evolutionary biology: inferences and implementation in R. EcoEvoRxiv. https://doi.org/10.32942/osf.io/hs38a

Hadfield, J.D. 2010. MCMC methods for multi-response generalized linear mixed models: the MCMCglmm R package. Journal of Statistical Software 33, 1–22.
